# Supplementary figures and images for: Multi-Omic Analysis of Two Common P53 Mutations: Proteins Regulated by Mutated P53 as Potential Targets for Immunotherapy
Source: Cancers (Basel). 2022 Aug 17;14(16):3975. doi: 10.3390/cancers14163975 (PMC9406384; doi:10.3390/cancers14163975)

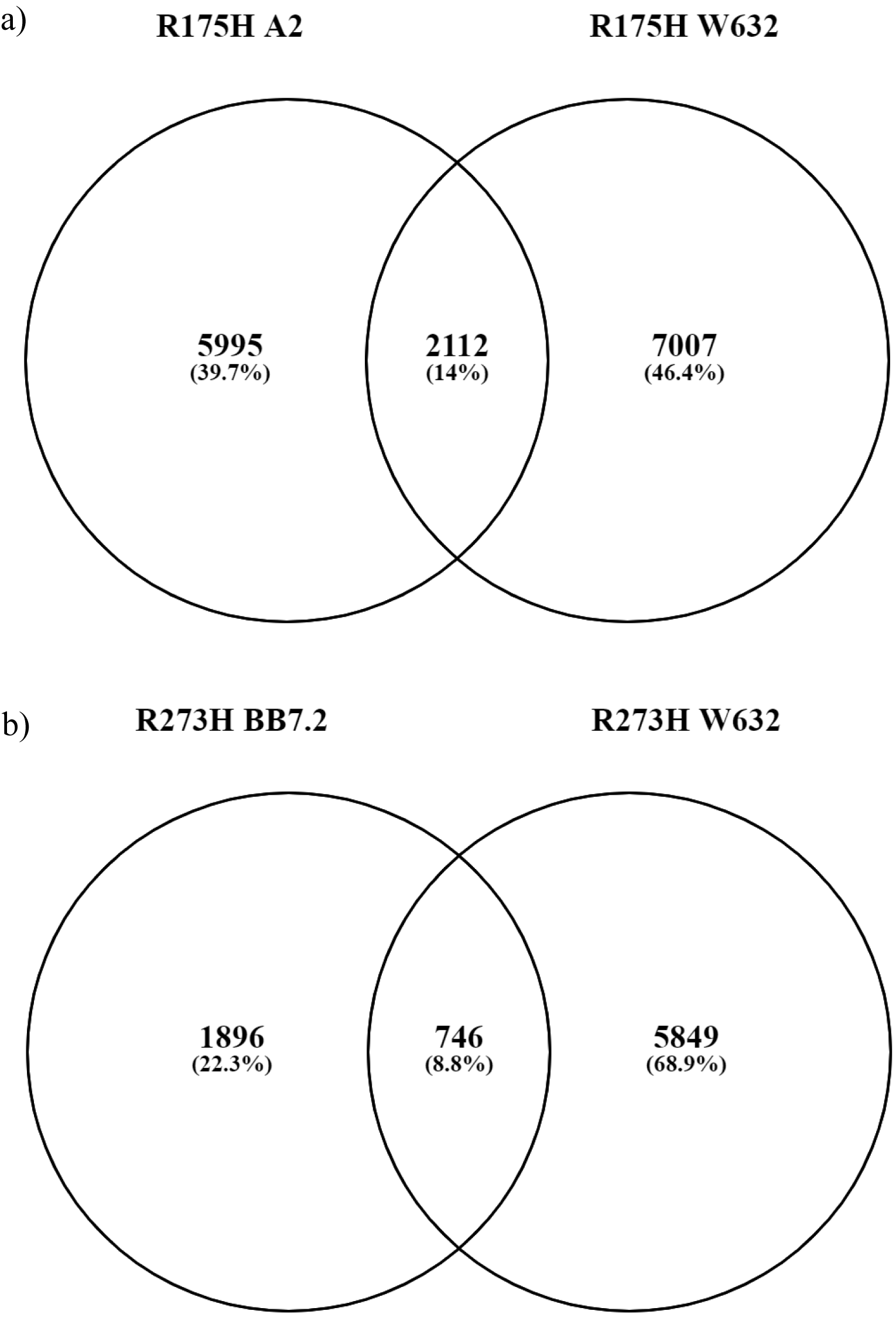

Supplement: Supplementary file 1 [file cancers-14-03975-s001.zip › Supplementary Figure S1.tif]

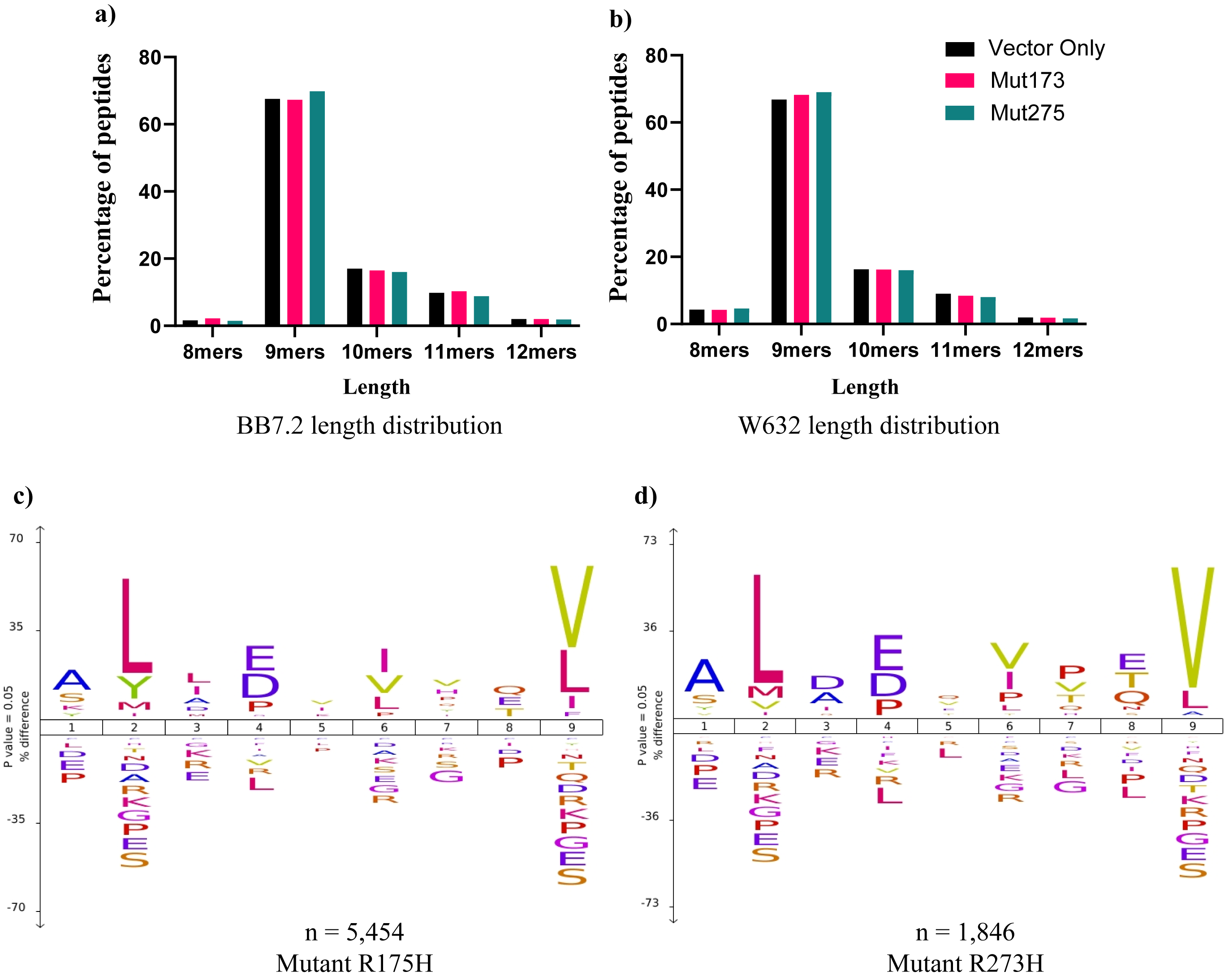

Supplement: Supplementary file 1 [file cancers-14-03975-s001.zip › Supplementary Figure S2.tif]

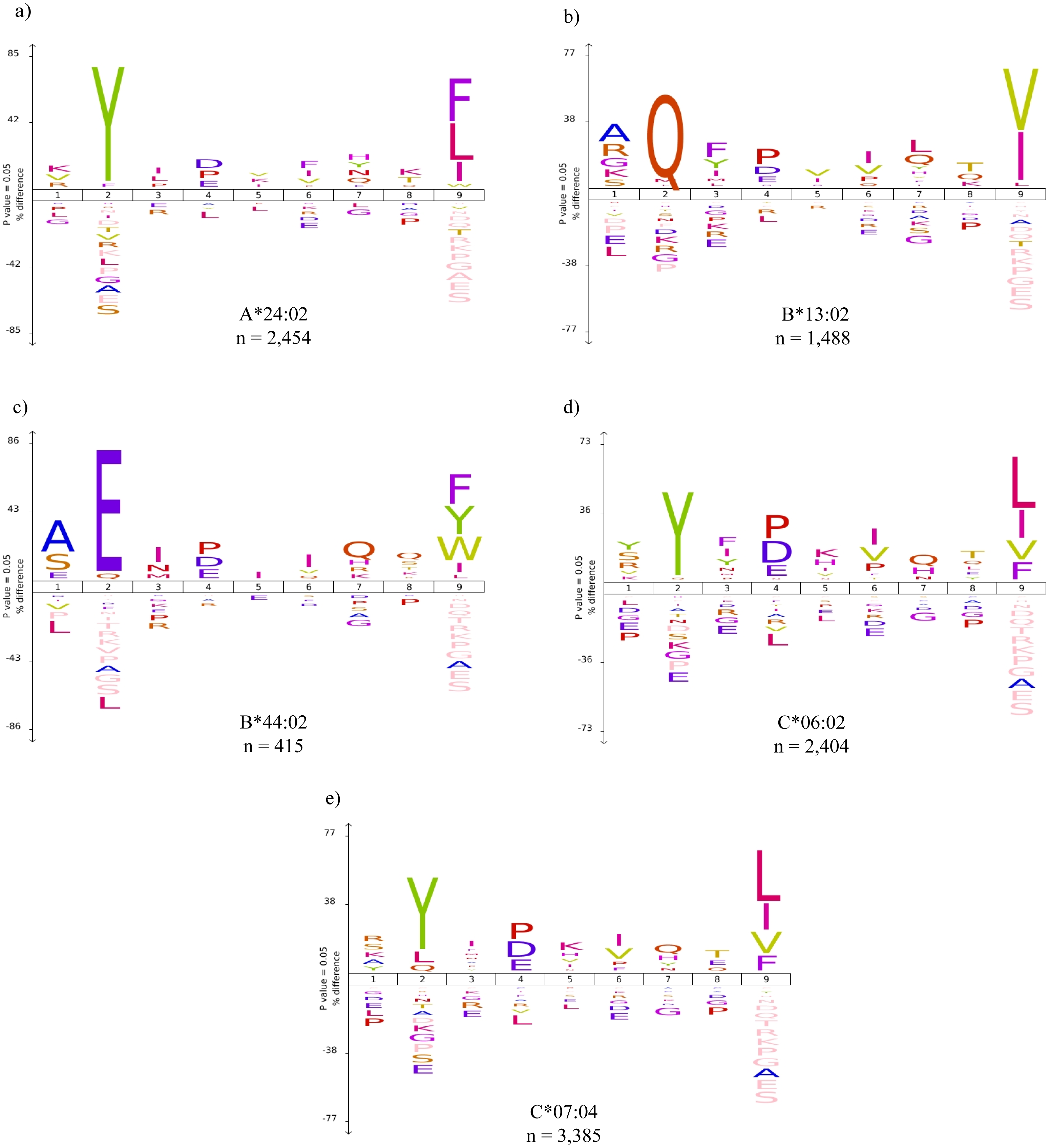

Supplement: Supplementary file 1 [file cancers-14-03975-s001.zip › Supplementary Figure S3.tif]
